# Partially defatted black soldier fly larva meal inclusion in piglet diets: effects on the growth performance, nutrient digestibility, blood profile, gut morphology and histological features

**DOI:** 10.1186/s40104-019-0325-x

**Published:** 2019-02-19

**Authors:** Ilaria Biasato, Manuela Renna, Francesco Gai, Sihem Dabbou, Marco Meneguz, Giovanni Perona, Silvia Martinez, Ana Cristina Barroeta Lajusticia, Stefania Bergagna, Luca Sardi, Maria Teresa Capucchio, Enrico Bressan, Andrea Dama, Achille Schiavone, Laura Gasco

**Affiliations:** 1 Department of Agricultural, Forest and Food Sciences, University of Turin, Largo P. Braccini 2, 10095 Grugliasco, (TO) Italy; 2 Department of Veterinary Sciences, University of Turin, Largo P. Braccini 2, 10095 Grugliasco, (TO) Turin Italy; 3 National Research Council, Institute of Science of Food Production, Largo P. Braccini 2, 10095 Grugliasco, (TO) Italy; 4 SDSV – Teaching Structure of Veterinary Medicine, University of Turin, Largo P. Braccini 2, 10095 Grugliasco, (TO) Italy; 5 Department of Animal Production, University of Murcia, Campus de Espinardo, 30071 Murcia, Spain; 6 Department of Animal and Food Science, Autonomous University of Barcelona, Cerdanyola del Vallès, 08193 Bellaterra, Barcelona Spain; 7 Veterinary Medical Research Institute for Piemonte, Liguria and the Valle D’Aosta, Via Bologna 148, 10154 Turin, Italy; 8 Department of Veterinary Medical Sciences, University of Bologna, Via Tolara di Sopra 50, 40064 Ozzano dell’Emilia, (BO) Italy

**Keywords:** *Hermetia illucens* L., Histopathology, Insect meal, Performance, Weaning pigs

## Abstract

**Background:**

The aim of this trial was to investigate the effects of different inclusion levels of a partially defatted black soldier fly (BSF, *Hermetia illucens* L.) larva meal on the growth performance, nutrient digestibility, blood profile, gut morphology and histological features of piglets. A total of 48 newly weaned piglets were individually weighed (initial body weight (IBW): 6.1 ± 0.16 kg) and randomly allocated to 3 dietary treatments (4 boxes as replicates/treatment and 4 animals/box). BSF larva meal was included at increasing levels (0% [BSF0], 5% [BSF5] and 10% [BSF10]) in isonitrogenous and isoenergetic diets formulated for two feeding phases: I (from d 1 to d 23) and II (from d 24 to d 61). The weight gain (WG), average daily gain (ADG), average daily feed intake (ADFI) and feed conversion ratio (FCR) were calculated for each feeding phase and for the whole trial. The haematochemical parameters and nutrient digestibility of the piglets were also evaluated. A total of 3 piglets per box were slaughtered on d 61 and the slaughtered piglets were submitted to morphometric investigations and histopathological examinations.

**Results:**

No overall significant differences were observed for growth performance (*P* > 0.05), except for the ADFI of phase II, which showed a linear response to increasing BSF meal levels (*P* < 0.05, maximum for the BSF10 group). Dietary BSF meal inclusion did not significantly influence the blood profile, except as far as monocytes and neutrophils are concerned, and these showed a linear and quadratic response, respectively, to increasing BSF meal levels (*P* < 0.05, maximum for the BSF10 and BSF5 groups, respectively). On the other hand, the nutrient digestibility, gut morphology and histological features were not affected by dietary BSF meal inclusion (*P* > 0.05).

**Conclusions:**

The obtained results show that a partially defatted BSF larva meal can be used as a feed ingredient in diets for weaned piglets without negatively affecting their growth performance, nutrient digestibility, blood profile, gut morphology or histological features.

## Background

A recent FAO forecast has indicated that the world will be inhabited by over 9.5 billion people by 2050 and that animal protein production will have to more than double to meet the increasing food demand [1]. Within the livestock sector, pigs constitute one of the fastest growing sub-sectors. Furthermore, as a result of changes in consumption patterns, the world’s pork meat production has increased from 95 to over 130 million tons in the last 15 years, and currently represents the second most consumed meat in the world [2]. Furthermore, it has been estimated that this production will increase by over 40% by 2050 [1]. Soybean meal (SBM) is the most frequently used protein source for pig compound feeds, with a recorded global world production of about 300 million tons in 2016 [3]. Compound feeds play a primary role in the world’s livestock production, with an overall annual amount of about 1 billion tons [4]. Plant proteins (e.g. SBM), fishmeal and other processed animal proteins have been used extensively in compounds feeds. However, these conventional sources are no longer considered sustainable and are limited by European regulations and/or increasing prices [5]. Therefore, a crucial challenge for future years is the discovery of new and sustainable protein sources for animal feeds.

Insects are one of the most promising novel protein sources [6–8], since they are rich in proteins and have an adequate amino acid profile, lipids and other valuable nutrients such as vitamins and minerals [6]. Moreover, it has been claimed that they are highly sustainable, as some species can be mass reared in organic streams at a much lower environmental cost than conventional protein sources [9, 10], thus exalting the concept of Circular Economy. Among the various suitable insects, black soldier fly (BSF) (*Hermetia illucens* L., HI) is one of the most promising species. It is a cosmopolitan and worldwide *Diptera* that belongs to the Stratiomyidae family [11]. BSF larvae can be reared on several organic substrates [12, 13] and can provide high-value meal that is rich in nutrients when processed [6]. Research on insect meals as animal feeds has increased rapidly, with promising results having been observed for poultry [14–16] and fish [17–19]. Dietary insect meal inclusion for pigs has also attracted increasing interest, with the authors’ attention being focused on growth performance [20–25], nutrient digestibility [23, 25, 26] and blood profile modifications [22, 23]. Recent studies have also investigated the potential gut health implications [24, 25] of using insects in swine diets, as a consequence of the increasing importance of this research topic in animal nutrition [27]. Within this scenario, insects could be particularly advantageous as a novel feed additive to improve gut health, because of the immune-system stimulating properties of chitin [28] and of the antibacterial/microbial activity of antimicrobial peptides [29] and lauric acid [25]. Spranghers et al. [25] have recently reported gut antimicrobial effects and unaffected gut morphology in weaned piglets fed BSF prepupae, but no information related to BSF larva utilization is currently available. Furthermore, there is still a lack of data about the potential diet-related, histopathological changes of organs that could be added to the knowledge on blood parameters in animal health assessments.

On the basis of the above reported background, the present study has been aimed at evaluating the effects of a partially defatted BSF larva meal on the growth performance, nutrient digestibility, blood profile, gut morphology and histological features of piglets, in order to increase knowledge and provide novel information on the potential use of insect meal in pig nutrition.

## Methods

The present trial was carried out at the Teaching Structure of Veterinary Medicine of the University of Turin (Italy).

### BSF larva meal and experimental diets

A partially defatted BSF larva meal, obtained by processing larvae reared on a vegetable by-products substrate, was purchased from Hermetia Deutschland GmbH & Co. KG (Baruth / Mark, Germany) and used in the trial. The BSF larva meal was partially defatted by means of a mechanical process which was performed using high pressure and without any solvents. No other information was provided by the producer regarding the substrate or processing methodologies, as this information is considered confidential. A total of 3 experimental diets were formulated to have the same net energy (NE) and crude protein (CP) levels. The diets for each dietary treatment were divided into 2 phases: phase I (days of trial: 1 to 23) and phase II (days of trial: 24 to 61). The diets were prepared to include, on a feed basis, increasing levels of BSF larva meal in substitution of 0, 30% and 60% (BSF0, BSF5 and BSF10, respectively) of SBM (Phase I) or 0, 32% and 65% (BSF0, BSF5 and BSF10, respectively) of SBM (Phase II). The diets were formulated to meet the nutrient requirements of piglets [30]. An aliquot of 0.5% of titanium oxide (TiO_2_ – Nova Chimica Srl) was added to all the diets to determine the nutrient digestibility at the end of the two feeding phases, using the index method reported by Zhang and Adeola [31]. Feed and fresh water were provided ad libitum. The ingredients of the experimental diets and their relative inclusion levels are reported in Table 1.

**Table 1:** Ingredients, proximate composition and net energy of the BSF larva meal and experimental diets

Items	BSF larva meal	Phase I (1–23 d)			Phase II (24–61 d)		
		BSF0	BSF5	BSF10	BSF0	BSF5	BSF10
Ingredients, g/kg as fed							
Corn meal	–	317.9	332.7	347.3	378.0	392.9	407.4
Barley meal	–	220.0	220.0	220.0	220.0	220.0	220.0
Soybean meal	–	200.0	140.0	80.0	185.0	125.0	65.0
Dried whey^a^	–	140.0	140.0	140.0	100.0	100.0	100.0
Wheat meal	–	60.0	60.0	60.0	60.0	60.0	60.0
Soybean oil	–	25.0	21.0	17.0	20.0	16.0	12.0
BSF meal^b^	–	0.0	50.0	100.0	0.0	50.0	100.0
Calcium carbonate	–	6.0	7.5	8.0	7.0	8.5	10.0
*L*-lysine	–	6.5	7.1	7.6	5.5	6.0	6.6
Titanium oxide	–	5.0	5.0	5.0	5.0	5.0	5.0
Premix piglets^c^	–	5.0	5.0	5.0	5.0	5.0	5.0
Dicalcium phosphate	–	5.0	3.0	0.0	6.0	3.0	0.0
*DL*-methionine	–	3.0	3.0	3.2	2.4	2.4	2.6
Salt	–	2.5	2.5	2.5	2.5	2.5	2.5
*L*-threonine	–	2.5	2.6	2.8	2.0	2.1	2.3
*L*-tryptophan	–	0.8	0.8	0.8	0.8	0.8	0.8
Pig aroma	–	0.8	0.8	0.8	0.8	0.8	0.8
Proximate composition, g/kg as fed^d^							
DM	947.4	899.8	903.1	906.4	894.0	897.3	900.7
Ash	76.7	47.2	46.4	45.5	46.1	45.3	44.6
CP	559.0	178.8	178.9	179.0	170.4	170.4	170.6
EE	85.0	73.5	73.4	73.3	61.9	61.8	61.7
Starch	–	346.8	354.3	361.7	384.2	391.8	399.1
NDF	192.3	109.8	115.2	120.7	114.2	119.6	125.1
ADF	87.0	40.5	42.1	43.6	41.4	43.0	44.5
Chitin	53.4	–	–	–	–	–	–
NE, MJ/kg	–	10.6	10.6	10.6	10.4	10.3	10.3
Calcium	8.2	5.1	5.1	5.2	5.5	5.6	5.6
Phosphorus	13.1	5.1	4.9	4.9	5.1	4.9	4.7
Magnesium	4.5	1.7	1.8	1.8	1.7	1.8	1.8
Sodium	1.1	2.1	2.1	2.2	1.8	1.8	1.9
Potassium	18.7	10.1	9.6	9.1	9.2	8.7	8.2
Chlorine	4.3	5.2	5.5	5.8	4.5	4.8	5.1
Manganese, mg/kg	203.0	66.8	75.2	83.5	66.8	75.1	83.5
Zinc, mg/kg	172.0	128.6	134.1	139.7	129.2	134.6	140.1
Iron, mg/kg	154.0	184.7	178.4	174.1	186.6	180.3	174.0
Essential amino acids, g/kg as fed^d^							
Methionine	7.3	5.5	5.5	5.7	4.9	4.8	5.0
Methione + Cysteine	12.0	8.4	8.2	8.3	7.7	7.5	7.5
Lysine	26.6	14.0	14.0	13.9	12.6	12.5	12.5
Threonine	20.1	9.0	9.0	9.1	8.2	8.2	8.2
Arginine	25.6	10.3	9.4	8.6	9.9	9.0	8.2
Valine	21.9	8.0	8.1	8.2	7.6	7.8	7.9
Tryptophan	34.8	2.9	2.9	2.8	2.8	2.7	2.7
Isoleucine	30.2	7.1	6.8	6.6	6.7	6.5	6.2
Leucine	14.4	13.7	13.2	12.7	13.3	12.8	12.4
Histidine	31.3	4.3	4.2	4.2	4.1	4.1	4.0
Phenylalanine	7.3	8.2	8.2	8.3	7.9	7.9	8.0

*BSF* black soldier fly; *DM* dry matter; *CP* crude protein; *EE* ether extract; *NDF* neutral detergent fiber; *ADF* acid detergent fiber; *NE* net energy; *BSF0* = control diet; *BSF5* = 5% inclusion level of black soldier fly meal; *BSF10* = 10% inclusion level of black soldier fly meal

^a^Added with 20% of coconut oil

^b^Black soldier fly larva meal purchased from Hermetia Deutschland GmbH & Co. KG (Baruth / Mark, Germany)

^c^Premix: 16,000 IU vitamin A; 2,000 IU vitamin D_3_; 75.0 mg vitamin E; 2.94 mg vitamin K_3_; 3.0 mg vitamin B_1_; 6.0 mg vitamin B_2_; 4.0 mg vitamin B_6_; 0.05 mg vitamin B_12_; 98 mg vitamin C; 21.0 mg pantothenic acid; 40.0 mg vitamin PP; 1.20 mg folic acid; 0.25 mg biotin; 1,500 IU 6-phytase; 700 IU xylanase; 312.5 IU glucanase; 145.68 mg copper; 0.05 mg cobalt; 0.44 mg selenium

^d^Values are reported as mean of duplicate analyses

### Chemical analyses of BSF larva meal and experimental diets

The BSF larva meal and the experimental diets were ground using a cutting mill (MLI 204; Bühler AG, Uzwil, Switzerland) and analysed to establish the dry matter (DM; AOAC# 934.01), CP (AOAC# 984.13), acid detergent fiber (ADF; AOAC# 973.18) and ash (AOAC# 942.05) contents according to AOAC International [32]. The ether extract (EE; AOAC# 2003.05) was analysed according to AOAC International [33]. The neutral detergent fiber (NDF) was analysed according to Van Soest et al. [34]; α-amylase (Sigma Aldrich, Saint Louis, MO, USA) was added, but no sodium sulphite, and the results were corrected for the residual ash content. All the analyses were performed in duplicate. The NE values of the diets were calculated according to Noblet et al. [35]. The amount of chitin of the BSF meal was estimated according to Finke [36]. Diet samples were prepared using a 22-h hydrolysis step in 6 mol/L HCl at 112 °C under a nitrogen atmosphere to determine amino acids (AA). Performic acid oxidation occurred prior to acid hydrolysis for methionine and cysteine. The AA in the hydrolysate were determined by means of HPLC, after post-column derivatization, according to the procedure described by Madrid et al. [37]. The proximate composition and NE of the BSF larva meal and of the experimental diets are shown in Table 1.

### Growth performance

A total of 48 commercial piglets (females, Topigs) were weaned at 20 ± 1 days of age, individually weighed (initial body weight [IBW]: 6.1 ± 0.16 kg) and randomly allotted to the 3 previously described dietary treatments. Each diet was replicated in 4 boxes of 4 piglets with a homogeneous IBW per box. The piglets – all identified with ear tags – were individually weighed at d 23 (feed change from phase I to phase II) and at the end of the trial (d 61). The trial lasted 40 d. The weight gain (WG), average daily gain (ADG), average daily feed intake (ADFI) and feed conversion ratio (FCR) were calculated for each feeding phase and for the whole experimental period. Mortality was checked daily and calculated as: (number of dead pigs / number of pigs at start) × 100.

### Nutrient digestibility

Faeces were collected for 4 consecutive days, twice a day (09:00 and 15:00). Sampling was conducted in each box for 2 consecutive hours (from 09:00 to 11:00 and from 15:00 to 17:00) as soon as the faeces were released by the piglets. The faeces of each sampling moment were pooled per box and immediately stored at − 18 °C. The feces samples were dried at 60 °C for 72 h, finely ground and pooled per box in equal proportions. Samples were analysed for DM, CP and EE, according to the AOAC methodologies described for feed analysis. The diet and feces samples were analysed to establish the TiO_2_ content using a UV spectrophotometer (UNICAN UV-Vis Spectrometry, Helios Gamma, United Kingdom) according to the method of Short et al. [38].

Using the analysed values for nutrient concentration, as well as the concentration of the TiO_2_ in the feed and faeces, the nutrient digestibility was calculated as follows: Digestibility (%): 100 - [100 × (Nf × Td) / (Nd × Tf)] where Nf and Nd are the nutrient concentrations (% DM) in the faeces and diet, respectively, and Td and Tf are the TiO_2_ concentrations (% DM) in the diets and faeces, respectively.

### Blood and serum parameters

A total of 12 piglets per treatment (3 animals per box) were randomly selected and slaughtered in the EC-approved slaughterhouse of SDSV – Teaching Structure of Veterinary Medicine of the University of Turin (Italy) at 61 days of age. The animals were stunned, by electrocution, and exsanguinated. Sterile tubes containing heparin were utilized to collect blood samples for haematological analysis, which was conducted using an automated laser cell counter calibrated for swine (MS4-S Hematology Analyzer, Melet Schloesing, Osny, France). A plain serum tube was instead utilized as a container to obtain serum for biochemistry analysis and protein electrophoresis, which was conducted using an automated photometer system (Screen Master Touch, Hospitex Diagnostics, Sesto Fiorentino, Florence, Italy) and a semi-automated agarose gel electrophoresis system (Sebia Hydrasys, Evry, France), respectively.

### Slaughter procedures and samples collection

The slaughtered piglets were submitted to anatomopathological investigations. Intestinal segment samples (approximately 5 cm in length) were excised and flushed with 0.9% saline to remove all the content. The collected segments of intestine were the tract after the pylorus (duodenum), the mid jejunum (jejunum) and the tract before the ileocecal junction (ileum). Samples of liver, spleen, lung, stomach and kidney were also collected. Gut segments and organs samples were fixed in Carnoy’s and 10% neutral buffered formalin (4% formaldehyde) solutions for morphometric analysis (gut segments) and a histopathological examination (gut segments and organs). Tissues were routinely embedded in paraffin wax blocks, sectioned at 5 μm thickness and mounted on glass slides.

### Histomorphological investigations

The Carnoy-fixed and paraffin-embedded intestinal sections were submitted to Haematoxylin & Eosin staining, and one slide per each intestinal segment was examined by means of light microscopy. Each slide was captured with a Nikon DS-Fi1 digital camera (Nikon Corporation, Minato, Tokyo, Japan), coupled to a Zeiss Axiophot microscope (Carl Zeiss, Oberkochen, Germany), using a 2.5× objective lens. NIS-Elements F software (Nikon Corporation, Minato, Tokyo, Japan) was used for image capturing. Morphometric analysis was performed by means of Image®-Pro Plus software, version 6.0 (Media Cybernetics Inc., Bethesda, Rockville, MD, USA). The evaluated morphometric indices were the villus height (Vh, from the top of the villus to the villus-crypt junction), crypt depth (Cd, from the villus-crypt junction to the base of the crypt) and the villus height to crypt depth ratio (Vh/Cd, calculated by dividing the villus height by the crypt depth) [39]. Morphometric measurements were performed on 10 well-oriented and intact villi and on 10 crypts chosen from the duodenum, jejunum and ileum [40].

Formalin-fixed and paraffin-embedded gut segments and organ sections were submitted to Haematoxylin & Eosin staining and examined by means of light microscopy. The observed histopathological alterations were evaluated using a semiquantitative scoring system as follows: absent (score = 0), mild (score = 1), moderate (score = 2) and severe (score = 3). A single score was given for the histopathological alterations of the liver, spleen, lung, stomach and kidneys, while the gut histopathological findings were assessed separately for the mucosa (inflammatory infiltrates) and submucosa (inflammatory infiltrates and gut-associated lymphoid tissue [GALT] activation) of each segment. The total score of each gut segment was obtained by adding the mucosa and submucosa scores, while the total score of each piglet was represented by the mean value of the duodenum, jejunum and ileum scores. All the slides were blind assessed by 3 observers and the discordant cases were reviewed, using a multi-head microscope, until unanimous consensus was reached.

### Statistical analysis

The data were analysed using the IBM SPSS Statistics V25.0.0 software (SPSS Inc., Chicago, IL, USA). An individual pig was used as the experimental unit to analyse the blood parameters, gut morphology and histological features (*n* = 12 per treatment), while each box was considered as the experimental unit for the growth performance and nutrient digestibility (*n* = 4 per treatment).

The growth performance, nutrient digestibility and blood parameter data were tested by means of one-way analysis of variance (ANOVA), and the effect of dietary BSF meal inclusion was evaluated by means of polynomial contrasts (linear and quadratic). The intestinal morphometric indices were analysed by fitting a general linear mixed model (GLMM). GLMM allowed the morphometric indices (Vh, Cd and Vh/Cd, separately) to depend on three fixed factors (diet, intestinal segment and the interaction between the diet and intestinal segment). Animal was included as a random effect to account for repeated measurements on the same piglet. The interactions between the levels of the fixed factors were evaluated by means of pairwise comparisons. The histopathological scores were analysed by means of the Kruskal-Wallis test (post-hoc test: Dunn’s Multiple Comparison test).

The results were expressed as the mean (growth performance, nutrient digestibility, blood parameter and histopathological data) or least square mean (intestinal morphometric indices) and standard error of the mean (SEM). *P* values ≤0.05 were considered statistically significant. A statistical trend was considered for 0.05 < *P* ≤ 0.10.

## Results

### Growth performance

The growth performance parameters of the piglets are summarized in Table 2. The piglets readily accepted the experimental diets, with a survival ranging from 98% (BSF10) to 100% (BSF0 and BSF5). Dietary BSF meal inclusion did not significantly influence the growth performance of the animals, except for the ADFI of phase II, which showed a linear response to increasing BSF meal levels (*P* < 0.05, with a maximum corresponding to the inclusion of 10% of BSF meal).

**Table 2:** Effects of dietary BSF larva meal inclusion on growth performance of the piglets (*n* = 4)

Items		Dietary treatments			SEM	*P*-value	
		BSF0	BSF5	BSF10		Linear	Quadratic
BW, kg	Initial	6.10	6.09	6.09	0.005	0.620	0.621
	End Phase I	12.06	12.00	12.34	0.191	0.576	0.643
	End Phase II	31.88	32.23	33.06	0.457	0.708	0.587
WG, kg	Phase I	5.96	5.91	6.24	0.190	0.574	0.656
	Phase II	19.81	20.23	20.72	0.498	0.399	0.894
	Total	25.78	26.14	26.96	0.581	0.361	0.791
ADG, kg/d	Phase I	0.26	0.26	0.27	0.008	0.574	0.656
	Phase II	0.52	0.53	0.55	0.013	0.399	0.894
	Total	0.42	0.43	0.44	0.010	0.361	0.791
ADFI, kg/d	Phase I	0.35	0.37	0.38	0.012	0.420	0.944
	Phase II	0.94	0.95	0.97	0.005	0.016	0.709
	Total	0.72	0.73	0.75	0.006	0.069	0.922
FCR	Phase I	1.35	1.42	1.41	0.030	0.578	0.479
	Phase II	1.81	1.79	1.76	0.038	0.718	0.976
	Total	1.71	1.70	1.70	0.033	0.848	0.888

*BSF* black soldier fly; *BW* body weight; *WG* weight gain; *ADG* average daily gain; *ADFI* average daily feed intake; *FCR* feed conversion ratio; *SEM* standard error of the mean; *BSF0* = control diet; *BSF5* = 5% inclusion level of black soldier fly meal; *BSF10* = 10% inclusion level of black soldier fly meal

### Nutrient digestibility

Dietary BSF meal inclusion did not significantly affect the piglets’ digestibility of the diets (Table 3).

**Table 3:** Effects of dietary BSF larva meal inclusion on nutrient digestibility in the piglets (*n* = 4, end of both feeding phases)

Items		Dietary treatments			SEM	*P*-value	
		BSF0	BSF5	BSF10		Linear	Quadratic
DM	Phase I	0.960	0.955	0.959	0.003	0.924	0.503
	Phase II	0.958	0.954	0.958	0.003	0.926	0.534
OM	Phase I	0.965	0.960	0.964	0.003	0.857	0.524
	Phase II	0.964	0.960	0.964	0.003	0.913	0.518
CP	Phase I	0.803	0.808	0.828	0.012	0.443	0.805
	Phase II	0.766	0.777	0.795	0.015	0.466	0.916
EE	Phase I	0.811	0.857	0.856	0.014	0.220	0.442
	Phase II	0.853	0.828	0.865	0.012	0.716	0.291

*BSF* black soldier fly; *DM* dry matter; *OM* organic matter; *CP* crude protein; *EE* ether extract; *SEM* standard error of the mean; *BSF0* = control diet; *BSF5* = 5% inclusion level 5% inclusion level of black soldier fly meal; *BSF10* = 10% inclusion level of black soldier fly meal

### Blood and serum parameters

The blood and serum parameters of the piglets are shown in Tables 4 and 5. Dietary BSF meal inclusion did not significantly influence the haematochemical profile or serum protein electrophoresis of the animals, except for the monocytes and neutrophils, which showed a linear and quadratic response, respectively, to increasing BSF meal levels (*P* < 0.05, with the maximum corresponding to the inclusion of 10% and 5% of BSF meal, respectively).

**Table 4:** Effects of dietary BSF larva meal inclusion on haematochemical parameters of the piglets (*n* = 12, end of the trial)

Items	Dietary treatments			SEM	*P*-value	
	BSF0	BSF5	BSF10		Linear	Quadratic
White blood cells, 10^6^/mm^3^	13.10	14.99	14.85	0.869	0.435	0.591
Lymphocytes, %	55.73	50.13	60.24	2.167	0.368 0.033	0.086 0.305
Monocytes, %	3.92	4.00	4.78	0.167		
Neutrophils, %	36.16	41.72	26.12	2.485	0.076	0.035
Eosinophils, %	3.81	3.78	8.39	0.801	0.078	0.299
Basophils, %	0.382	0.383	0.30	0.041	0.421	0.630
Red blood cells, 10^6^/mm^3^	7.57	7.69	7.89	0.306	0.681	0.953
MCV, fL	57.35	57.23	57.40	0.354	0.948	0.849
Haematocrit, %	43.43	43.91	45.47	1.812	0.658	0.892
MCH, pg	18.17	18.76	19.35	0.366	0.204	0.997
MCHC, g/dL	31.78	32.90	33.90	0.687	0.224	0.968
RDW, fL	14.13	14.15	14.15	0.159	0.956	0.974
Haemoglobin, g/dL	13.81	14.39	14.77	0.562	0.506	0.932
Platelets, 10^6^/mm^3^	197.18	195.00	181.42	11.946	0.602	0.827
MPV, fL	11.98	11.74	12.10	0.081	0.518	0.081
Procalcitonin, %	0.24	0.23	0.22	0.015	0.598	0.978
PDW, fL	8.56	8.14	7.81	0.323	0.366	0.955
GOT, IU/L	111.21	126.78	143.31	12.808	0.324	0.986
GPT, IU/L	59.73	63.15	55.38	2.068	0.380	0.206
ALP, IU/L	28.52	31.19	60.49	5.952	0.082	0.880
GGT, IU/L	43.49	48.30	40.26	2.463	0.589	0.223
Total protein, g/dL	6.67	6.02	5.98	0.194	0.163	0.460
Phosphorus, mg/dL	14.65	17.10	14.94	0.966	0.929	0.272
Iron, μg/dL	118.39	146.99	130.32	8.975	0.617	0.242
Magnesium, mEq/L	1.65	2.09	1.93	0.116	0.335	0.230
Uric acid, mg/dL	1.66	3.03	2.71	0.576	0.481	0.502
Cholesterol, mg/dL	52.31	55.41	51.30	2.647	0.869	0.533
Triglycerides, mg/dL	58.93	47.94	55.28	4.444	0.764	0.342
Creatinine, mg/dL	1.44	1.68	1.61	0.058	0.242	0.212

*BSF* Black soldier fly; *MCV* mean corpuscular volume; *MCH* mean corpuscular haemoglobin; *MCHC* mean corpuscular haemoglobin concentration; *RDW* red blood cell distribution width; *MPV* mean platelet volume; *PDW* platelet distribution width; *GOT* glutamic oxaloacetic transaminasi; *GPT* glutamate-pyruvate transaminasi; *ALP* alkaline phosphatase; *GGT* gamma-glutamyl transferase; *SEM* standard error of the mean. *BSF0* = control diet; *BSF5* = 5% inclusion level 5% inclusion level of black soldier fly meal; *BSF10* = 10% inclusion level of black soldier fly meal

**Table 5:** Effects of dietary BSF larva meal inclusion on serum protein electrophoresis of the piglets (*n* = 12, end of the trial)

Items	Dietary treatments			SEM	*P*-value	
	BSF0	BSF5	BSF10		Linear	Quadratic
Albumin, %	45.73	46.41	47.50	0.372	0.056	0.789
Albumin, g/dL	3.07	2.79	2.84	0.092	0.335	0.398
Alpha globulin, %	25.00	25.63	25.01	0.280	0.988	0.307
Alpha globulin, g/dL	1.67	1.55	1.50	0.054	0.209	0.746
Beta globulin, %	17.02	16.43	16.41	0.186	0.198	0.467
Beta globulin, g/dL	1.13	0.99	0.98	0.033	0.080	0.307
Gamma globulin, %	12.26	11.53	11.08	0.381	0.227	0.868
Gamma globulin, g/dL	0.80	0.70	0.66	0.034	0.106	0.673

*BSF* black soldier fly; *SEM* standard error of the Smean; *BSF0* = control diet; *BSF5* = 5% inclusion level 5% inclusion level of black soldier fly meal; *BSF10* = 10% inclusion level of black soldier fly meal

### Histomorphological investigations

The effects of the diet, intestinal segment and interaction between the diet and intestinal segment on the intestinal morphometric indices of the piglets are summarized in Table 6. The intestinal segment significantly affected Vh, Cd and Vh/Cd (*P* < 0.001, *P* < 0.05 and *P* < 0.01, respectively). The duodenum and jejunum showed higher Vh and Vh/Cd (*P* < 0.01 and *P* < 0.05, respectively) than the ileum, while a greater Cd (*P* < 0.05) was observed in the duodenum than in the other gut segments. On the contrary, there was no influence of diet or interaction between the diet and intestinal segment on the morphometric indices.

**Table 6:** Intestinal morphometric indices in the piglets in relation to diet and intestinal segment (*n* = 12, end of the trial)

Items	Diet (D)			Intestinal segment (IS)			SEM	*P*-value		
	BSF0	BSF5	BSF10	Duodenum	Jejunum	Ileum		D	IS	D × IS
Vh, mm	0.38	0.36	0.38	0.41^a^	0.38^a^	0.34^b^	0.012	0.371	< 0.001	0.709
Cd, mm	0.11	0.11	0.10	0.12^a^	0.10^b^	0.11^b^	0.003	0.256	0.009	0.999
Vh/Cd	3.47	3.35	3.72	3.64^a^	3.71^a^	3.19^b^	0.126	0.159	0.007	0.566

*BSF0* = control; *BSF5* = 5% inclusion level 5% inclusion level of black soldier fly meal; *BSF10* = 10% inclusion level of black soldier fly meal; *Vh*, villus height; *Cd*, crypt depth; *Vh/Cd*, villus height to crypt depth ratio

The means with different superscript letters (a, b) within the same row per fixed effect (i.e. diet, intestinal segment) differ significantly (*P* < 0.05)

**Fig. 1:**
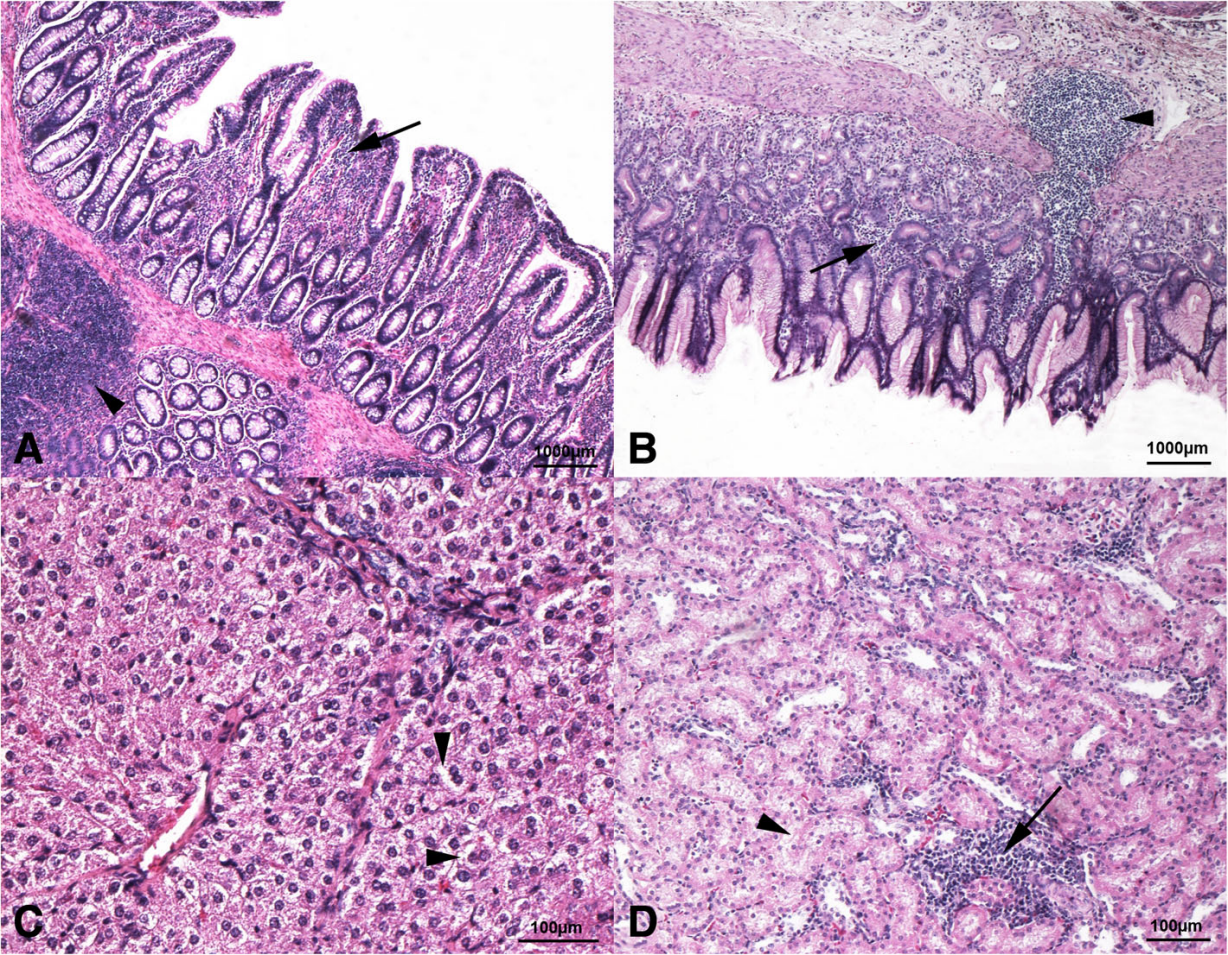
Histopathological findings of the piglets. **a** BSF0 group. Mild and multifocal mucosal lymphoplasmacytic inflammation (arrow), as well as focal and moderate gut-associated lymphoid tissue (GALT) activation (arrowhead), are observed in the ileum. 5× haematoxylin-eosin stain. **b** BSF5 group. Stomach shows mild and multifocal mucosal lymphoplasmacytic inflammation (arrow) along with focal and moderate GALT activation (arrowhead). 5× haematoxylin-eosin stain. **c** BSF10 group. Liver, centrolobular zone. Moderate and multifocal vacuolar degeneration of the hepatocytes (arrowheads) is identified. 20× haematoxylin-eosin stain. **d** BSF0 group. Moderate and multifocal interstitial lymphoplasmacytic inflammation (arrow) and tubular steatosis (arrowhead) are observed in the kidneys. 10× haematoxylin-eosin stain

Histopathological alterations developed in the stomach, gut, liver and kidneys of the piglets for all the dietary treatments (Fig. 1). On the contrary, the spleen and lung showed no alterations. The stomach and gut showed mucosal/submucosal lymphoplasmacytic or eosinophilic inflammation, with or without GALT activation. Vacuolar degeneration of the hepatocytes and lymphoplasmacytic inflammation with or without fibrous tissue deposition was observed in the liver. Finally, interstitial lymphoplasmacytic inflammation and tubular steatosis were identified in the kidneys. However, dietary BSF meal inclusion did not affect the severity of the observed histopathological alterations (Table 7).

**Table 7:** Effects of dietary BSF larva meal inclusion on the histopathological scores of the piglets (*n* = 12, end of the trial)

Items	BSF0	BSF5	BSF10	SEM	*P*-value
Stomach	1.17	1.06	1.33	0.122	0.770
Gut	2.79	3.26	3.08	0.26	0.933
Liver	0.64	0.53	0.68	0.115	0.576
Kidney	0.82	0.96	1.00	0.064	0.381
Spleen	Absence of alterations Absence of alterations				
Lung					

*BSF*, black soldier fly; *SEM*, standard error of the mean; *BSF0* = control diet; *BSF5* = 5% inclusion level 5% inclusion level of black soldier fly meal; *BSF10* = 10% inclusion level of Black Soldier Fly meal

The data are expressed as the mean of the scores (0 = absence of alterations; 1 = mild alterations; 2 = moderate alterations; 3 = severe alterations). The gut scores resulted from the mean of the three gut segments scores (in turn obtained from the adding of the mucosa and submucosa scores)

## Discussion

### Growth performance

Overall, dietary BSF meal inclusion did not influence the growth performance of the piglets involved in the present study, although an increasing ADFI was identified in phase II for increasing levels of BSF meal inclusion. This could be attributed to an increased diet palatability, related to insect meal inclusion, as already reported by Jin et al. [23]. Furthermore, the higher feed assumption was only observed in the second phase of the trial, thus suggesting a potential tendency of piglets to eat the insect-based diets. However, the overall growth performance observed in the current research is in agreement with Driemeyer [22], who showed no effects for the growth performance of piglets fed maize-soybean and fish meal-based diets in which the full-fat BSF inclusion level was 3.5% in partial substitution of fish meal. Similarly, Spranghers et al. [25] have recently observed no effect on the growth performance of weaned piglets fed diets including full-fat (4% and 8%) and defatted (5.4%) BSF prepupae. More heterogeneous results were previously reported, when other insect larvae were used as protein sources in swine feeds. In fact, piglets fed diets in which HI replaced 75% of SBM tended to show a slightly lower growth, feed intake and feed and protein conversion ratio than those fed a control diet [24]. Jin et al. [23] also found an improved growth performance in weaning pigs fed corn-soybean meal-based diets in which increasing levels of a full-fat *Tenebrio molitor* (TM) larva meal (from 1.5% to 6.0%) was used to partially replace soybean meal and soy oil, and they attributed this outcome to an increased palatability of yellow mealworm. Chen et al. [41] also observed similar results for increasing dietary TM protein concentrates of up to 6%. Improved weight gain was also found in weaning pigs fed *Musca domestica* (L.) [42] and *Bombyx mori* (L.) [43] meal inclusion diets. On the other hand, dietary house-fly larva meal inclusion has not been reported to influence the growth performance of sows and their offspring [20] or early weaned piglets [21]. The heterogeneity of the results obtained from these previous studies may be related to the nutritive value of the insect meal that was used, which could have been influenced by the species, the insect life stage (adult, larva or pupa) and the insect rearing substrate [7].

### Nutrient digestibility

Dietary BSF meal inclusion did not influence the nutrient digestibility of the piglets in the present study. This result is in agreement with the results of Spranghers et al. [25], who observed a similar apparent total tract digestibility of nutrients in BSF prepupa- and basal diet-fed weaned piglets. On the contrary, Newton et al. [26] reported lower DM and higher EE digestibility in piglets fed 33% dried HI larva meal than those fed soybean meal, although the CP digestibility was similar. With regards to other insect species, Jin et al. [23] observed a linear improvement in DM and CP digestibility for increasing levels of dried mealworm in diets for weaning pigs. As already hypothesized for growth performance, these discrepancies may be related to the different insect species and life stages, dietary inclusion levels and fat extraction methods. Furthermore, the heterogeneity of the feed ingredients used in the present and above-mentioned researches does not allow any reliable statement to be made about the effective role of BSF in nutrient digestibility modifications. However, the identification of unaffected or even improved nutrient digestibility may reflect a species-specific ability of chitin digestion, as suggested by the high distribution and activity of chitinase [44] and positive selection of chitinase genes [45] previously reported in pigs. Feeding chito-oligosaccharide to weaning piglets has also been reported to improve DM and crude fat digestibility [46]. Finally, no data about organic matter digestibility have been reported for insect-fed pigs, thus making the values obtained in the present study the first ones available.

### Blood and serum parameters

 All the haematochemical parameters recorded for the piglets in the present study fell within the physiological reference intervals reported for swine [47], thus suggesting that the insect meal utilization did not negatively influence the health status of the animals. Moreover, the dietary BSF meal inclusion did not significantly affect the blood and serum traits of the pigs in the current research, although increasing monocytes and neutrophils were identified for increasing levels of BSF meal. This finding appears difficult to explain, since none of the BSF piglets showed any signs of physical distress or inflammatory diseases. However, it is important to underline that the haematological and biochemical profiles of herds have rarely been used in pig veterinary practice. Therefore, further studies are recommended to assess their value as a diagnostic technique [22]. Similar findings, in terms of unaffected blood haematology and biochemical parameters (albumin, calcium, phosphorus, iron, IgA, IgG and IgM), have been observed in piglets fed diets with a full-fat 3.5% BSF inclusion level [22]. Jin et al. [23] instead reported that the dietary supplementation of up to 6% of dried full-fat mealworm in weaning pigs decreased blood urea nitrogen and increased the serum insulin-like growth factor. However, no significant effects were observed for the serum IgG and IgA concentrations.

### Histomorphological investigations

Dietary BSF meal inclusion did not influence the gut morphology of the piglets in the present study. This result is in agreement with that of Spranghers et al. [25], who observed no effect on the jejunal morphology of BSF prepupa-fed weaned piglets. It is well known that integrity of the intestinal structure is required for the maintenance of intestinal nutrition [48]. Furthermore, modifications of gut morphometric features have been reported to determine significant changes in the weight gain of piglets [40, 49–51]. Since both the intestinal morphology and the growth performance of the BSF-fed piglets in the current research were unaffected, it is reasonable to hypothesize that insect meal utilization does not negatively influence gut development or nutrient absorption. Another interesting aspect that should be considered is that, independently of dietary BSF meal inclusion, the duodenum and jejunum showed greater morphometric indices than the ileum. Despite the heterogeneous information about the morphological asset of the different gut segments currently available in literature [52], the identification of higher morphometric values in the duodenum and jejunum appears to be rather constant for pigs [53, 54]. Therefore, the findings of the present study confirm what was previously reported in literature.

Dietary BSF meal inclusion did not affect the development or severity of the histopathological alterations in the piglets in the current research. Gastric inflammation was attributed to the sensitiveness of pigs to damage of the gastric mucosa, which frequently occurs as a consequence of stress and feeding practices [55]. An analogous consideration can also be made for gut inflammation. The hepatic and renal alterations varied from mild to moderate and from focal to multifocal in all the pigs, thus suggesting no clinical relevance. Furthermore, no alteration of the serum chemistry was observed in relation to the liver and renal functions.

## Conclusions

The present study suggests that a dietary inclusion of up to 10% of partially defatted BSF larva meal could be used for weaning pigs without any adverse effects on the growth performance, blood profile, nutrient digestibility, gut morphology or histological features. These results confirm that BSF has the potential to become a valuable source of sustainable proteins in pig nutrition. However, since the price of insects is still not competitive, future studies should be focused on the use of BSF as a potential feed additive to improve the gut health of pigs, in particular through the modulation of the gut microbiota and mucin composition.

## Abbreviations

AA: Amino acids
ADF: Acid detergent fiber
ADFI: Average daily feed intake
ADG: Average daily gain
ALP: Alkaline phosphatase
BSF: Black soldier fly
BW: Body weight
Cd: Crypt depth
CP: Crude protein
DE: Digestible energy
DM: Dry matter
EE: Ether extract
FBW: Final body weight
FCR: Feed conversion ratio
GGT: Gamma-glutamyl transferase
GOT: Glutamic oxaloacetic transaminase
GPT: Glutamate-pyruvate transaminase
HI: *Hermetia illucens*
IBW: Initial body weight
MCH: Mean corpuscular haemoglobin
MCHC: Mean corpuscular haemoglobin concentration
MCV: Mean corpuscular volume
MPV: Mean platelet volume
NDF: Neutral detergent fiber
NE: Net energy
PDW: Platelet distribution width
RDW: Red blood cell distribution width
SBM: Soybean meal
SEM: Standard error of the mean
TM: *Tenebrio molitor*
Vh: Villus height
Vh/Cd: Villus height to crypt depth ratio
WG: Weight gain
